# Emergence and significance of carbohydrate-specific antibodies

**DOI:** 10.1038/s41435-020-0105-9

**Published:** 2020-08-05

**Authors:** Katharina Kappler, Thierry Hennet

**Affiliations:** Institute of Physiology, University of Zurich, Zurich, Switzerland

**Keywords:** Antibodies, Antimicrobial responses

## Abstract

Carbohydrate-specific antibodies are widespread among all classes of immunoglobulins. Despite their broad occurrence, little is known about their formation and biological significance. Carbohydrate-specific antibodies are often classified as natural antibodies under the assumption that they arise without prior exposure to exogenous antigens. On the other hand, various carbohydrate-specific antibodies, including antibodies to ABO blood group antigens, emerge after the contact of immune cells with the intestinal microbiota, which expresses a vast diversity of carbohydrate antigens. Here we explore the development of carbohydrate-specific antibodies in humans, addressing the definition of natural antibodies and the production of carbohydrate-specific antibodies upon antigen stimulation. We focus on the significance of the intestinal microbiota in shaping carbohydrate-specific antibodies not just in the gut, but also in the blood circulation. The structural similarity between bacterial carbohydrate antigens and surface glycoconjugates of protists, fungi and animals leads to the production of carbohydrate-specific antibodies protective against a broad range of pathogens. Mimicry between bacterial and human glycoconjugates, however, can also lead to the generation of carbohydrate-specific antibodies that cross-react with human antigens, thereby contributing to the development of autoimmune disorders.

## Structural diversity of carbohydrate antigens

Despite their prominent occurrence at the surface of all cells and virus particles, carbohydrates do not elicit immune responses like peptide antigens. Yet, carbohydrate-specific antibodies are widespread among all classes of immunoglobulins [1]. Carbohydrate antigens eliciting an immune response represent structures consisting of monosaccharides and oligosaccharides that are foreign to the host. Although human glycoconjugates encompass a tremendous diversity of structures, human glycosylation is based on the combination of only the ten monosaccharides glucose (Glc), galactose (Gal), N-acetylglucosamine (GlcNAc), N-acetylgalactosamine (GalNAc), glucuronic acid, iduronic acid, xylose, mannose, fucose (Fuc), and the sialic acid N-acetylneuraminic acid (NeuAc) [2]. By comparison, bacterial glycosylation is based on an alphabet consisting of more than one hundred distinct monosaccharides. In addition to the ten monosaccharides found on human cells, bacterial glycans contain several deoxysugars and deoxyaminosugars, such as rhamnose, quinovose, N-acetylrhamnosamine and N-acetylquinovosamine, arabinose and 3-deoxy-D-manno-octulosonic acid (KDO) [3, 4]. In contrast, Fuc is the only deoxyhexose [5] and NeuAc the only sialic acid [6] found on human glycoconjugates. Beyond monosaccharide composition, carbohydrate conformations and thereby antigenic properties largely depend on the types of glycosidic linkages connecting monosaccharides. Accordingly, Glc can be recognized as a foreign antigen and elicit the production of antibodies, when it is polymerized through linkages unused in human cells, such as β1–3 or β1–6 found in fungal and bacterial β-glucans [7]. The multitude of combinations of monosaccharides together with a wide range of glycosidic linkages occurring in prokaryotes [8] and eukaryotes [4] yield an extensive repertoire of carbohydrate antigens susceptible to stimulate the production of antibodies in humans.

### Commonly recognized carbohydrate antigens

**Fig. 1:**
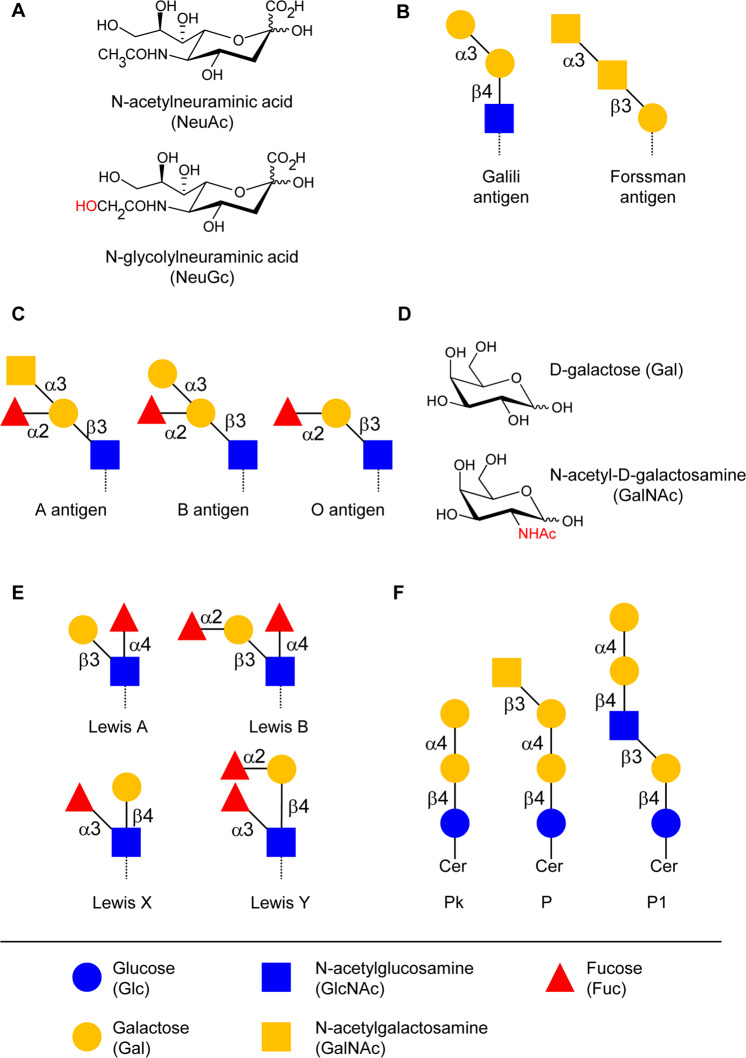
Commonly recognized glycan epitopes by human antibodies. **a** N-acetyl-neuraminic acid (NeuAc) and N-glycolyl-neuraminic acid (NeuGc) differ only by the occurrence of an additional hydroxyl group in NeuGc. **b** Schematic structure of Forssman and Galili antigen. Glycosidic linkages are marked using the minimal nomenclature; α3 for α1–3, β3 for β1–3 and β4 for β1–4. **c** Schematic structure of ABO blood group antigens. **d** Chemical composition of galactose (Gal) and N-acetylgalactosamine (GalNAc) with highlighted acetamido group at C2. **e** Schematic structure of Lewis antigens Lewis A, B, X, and Y. **f** Structure of the P blood group antigens Pk, P, and P1.

A large pool of serum IgM and IgG recognizes a variety of carbohydrate antigens [9–12]. These most prominently recognized antigens include the monosaccharides α-rhamnose, α-GlcNAc and β-GlcNAc [10], and the sulfated Gal(β1–4)GlcNAc structure [9]. Antibodies against β4-linked oligosaccharides of Glc, α-Gal and GlcNAc(β1–4)GlcNAc are also commonly observed [10]. The repertoires of carbohydrate antigens recognized show a large inter-individual variability among human beings [10, 13]. Despite the recognition of mono- and disaccharide epitopes, most circulating carbohydrate-specific antibodies bind with low specificity to larger glycoconjugates, thus preventing the occurrence of disseminated antibody-mediated inflammatory reactions and autoimmunity [9]. α-Rhamnose is a monosaccharide antigen associated with high antibody titer in human serum [14]. This prominence is explained by the absence of rhamnose on human glycoconjugates and its widespread occurrence on microbial polysaccharides [15, 16]. Another human xenoantigen associated with carbohydrate-specific antibodies is the sialic acid N-glycolylneuraminic acid (NeuGc). Through the inactivation of the cytidine-monophosphate-N-acetylneuraminic acid hydroxylase gene, humans have lost the ability to produce NeuGc besides NeuAc [17]. The contact to glycoproteins containing NeuGc stimulates the production of high antibody titers toward NeuGc [18–20]. Antibodies specific for NeuGc do not cross-react with NeuAc despite the close structural similarity between both sialic acids [21] (Fig. 1a).

The two disaccharide structures Gal(α1–3)Gal and GalNAc(α1–3)GalNAc are also major epitopes recognized by carbohydrate-specific antibodies. The former disaccharide is commonly referred to as the Galili or α-Gal epitope [22] (Fig. 1b). α-Gal antibodies [23] make up 1% of circulating IgG in human serum [24, 25]. α-Gal antibodies are only present in the blood of humans, apes, and old-world monkeys, because these taxa have an inactive pseudogene instead of a functional α1–3 Gal-transferase gene [26]. The elevated titers of circulating antibodies targeting Gal(α1–3)Gal explains the hyperacute rejection of grafted xenotransplants, such as pig organs, in humans [27, 28]. α-Gal antibodies develop during the first 2 years of life [29], following the exposure to intestinal bacteria expressing the Galili epitope on their cell wall glycoconjugates. The disaccharide GalNAc(α1–3)GalNAc, also called Forssman antigen [30], is another epitope that is absent from human glycans but widespread on animal cells and on bacterial glycoconjugates (Fig. 1b). Accordingly, the Forssman antigen is the carbohydrate structure associated with the highest antibody titers in humans [31, 32].

Carbohydrate-specific antibodies also include antibodies targeting polymorphic oligosaccharide structures in human beings. Various blood group systems, such as the ABO, Lewis, and P antigen systems, are based on the selective expression of polymorphic glycosyltransferase genes. The *ABO* glycosyltransferase gene on human chromosome 9 comprises multiple alleles that encode either an α1–3 GalNAc-transferase yielding the A-antigen, or an α1–3 Gal-transferase yielding the B-antigen, or an inactive protein yielding the O-antigen [33] (Fig. 1c). The structural difference between the A and B antigens solely relates to the differential exchange of a hydroxyl group by an acetamido group at C2 (Fig. 1d), yet this difference and the presence of high titers of circulating antibodies against ABO antigens precludes the transfusion with ABO-incompatible blood. Two genes on chromosome 19 are responsible for the expression of Lewis antigens in endodermal tissue, such as intestinal epithelial cells, and secretions [34]. *FUT3* encodes an α1–3/1–4 Fuc-transferase, yielding the Lewis A antigen and *FUT2* encodes an α1–2 Fuc-transferase, which adds a second Fuc residue to the Lewis A antigen, yielding the Lewis B antigen [35] (Fig. 1e). Subsequent surface presentation of Lewis A and B antigens on erythrocytes is based on the transport and incorporation of antigen-expressing glycolipids into the cell membrane. Lewis X and Lewis Y antigens, which are not defined as blood cell antigens, are synthesized by the same glycosyltransferases using a different precursor glycan [34] (Fig. 1e). The P blood group system is defined by the presence of three major glycosphingolipid antigens, P1, P, and Pk, on human erythrocytes, resulting in five phenotypes. The two most common ones are P1 phenotype, expressing P1, P, and Pk antigens and P2 phenotype, expressing P1 and Pk antigens [36]. The *A3GALT* gene on chromosome 22 encodes an α1–4 Gal-transferase, which adds Gal to paragloboside or lactosylceramide, resulting in P1 and Pk antigens, respectively [37, 38]. The P antigen is produced by the *B3GALNT1* gene, yielding a β1–3 GalNAc-transferase 1, which adds a GalNAc to the Pk antigen [39] (Fig. 1f).

Carbohydrate-specific antibodies can be detected already early in life without immunization, as through infections and vaccinations. Antibodies occurring without prior immunization are often classified as natural antibodies [40, 41]. The early colonization of the gut by bacteria right after birth [42] exposes the immune system to a wide range of novel carbohydrate antigens, which leads, for example, to the emergence of α-Gal and ABO-specific antibodies [25, 43]. Thus, an early immune stimulation by commensal bacteria could lead to the production of carbohydrate-specific antibodies. The question arises whether all antibodies classified as natural antibodies are indeed non-antigen induced antibodies.

## Natural antibodies

In contrast to antigen-specific antibodies, which are produced in a T-cell-dependent manner by mature B cells, natural antibodies are defined as pre-immune antibodies, generated without antigenic stimulation and T-cell assistance [40]. Thus, natural antibodies are not strictly speaking antigen-specific, yet they contribute to protection from bacterial and viral infections by poly-reactive binding to a wide range of microbes [44, 45]. Natural antibodies, mainly comprising antibodies of the IgM class but also IgA [46] and IgG [47, 48], show low-binding affinities and occur in small amounts [40, 49]. Although natural antibodies do not undergo somatic hypermutation, a fraction of them may carry mutated variable regions, given that a low rate of hypermutation takes place even without T-cell signals [50, 51]. Natural antibodies are produced by innate-like B cells, mainly B1 CD5^+^ cells, upon activation of Toll-like receptors [40, 41, 52].

B1 cells are defined through the combined markers CD20^+^CD27^+^CD43^+^CD70^−^ [53] and can be CD5^+^ or CD5^−^ [54, 55]. CD5^+^ B1 cells, also called B1a cells, are mainly produced during fetal and neonatal development from progenitors in the fetal omentum or the fetal liver, while they are generally absent in adult bone marrow. CD5^−^ B1 cells, referred to as B1b cells, are also present in fetal omentum and liver, but additionally occur in adult bone marrow, thus providing persistent maintenance of the B1-cell pool [40, 54]. Conventional B cells, defined as B2 cells, in contrast, are absent from the fetal omentum. B1a cells and are typically encoded by germline V genes during VDJ recombination without or with a low rate of somatic hypermutation and low N-region diversity [54, 56–58]. Recent studies, however, highlighted the existence of mouse B1a cells, which produce antibodies with higher N-region diversity and antibodies that underwent somatic hypermutation and class switching with increasing age [59, 60]. As the levels of natural IgA and IgG but not of IgM remain low in germ-free mice [61], exposure to the gut microbiota, either through stimulation of innate immunity or through direct antigen stimulation, likely contributes to the emergence of natural IgG and IgA [40]. This explanation, however, would imply that not all natural antibodies adhere to the conventional definition as being antigen-independent.

## Development of carbohydrate-specific antibodies

Despite the broad occurrence and the large variety of carbohydrate-specific antibodies, surprisingly little is known about their origin and maturation. Carbohydrate-specific antibodies are traditionally believed to be induced in a T-cell-independent manner. For many years, the accepted dogma stated that carbohydrate-specific antibodies feature low affinity and specificity and are mainly confined to the IgG2 subclass in the blood [62–65]. However, recent studies described carbohydrate-specific antibodies among multiple immunoglobulin subclasses [66, 67] and demonstrated the existence of high-affinity carbohydrate-specific antibodies [68].

**Fig. 2:**
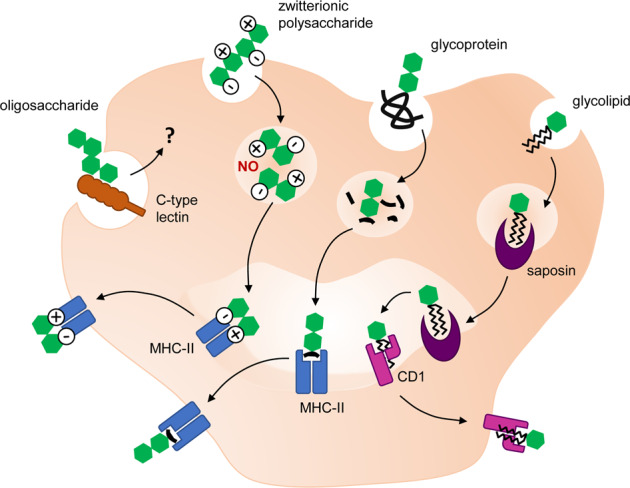
Antigen processing and presentation for different types of carbohydrate structures. Extracellular glycoproteins are engulfed in endocytic or phagocytic vesicles, broken down in phagolysosomes and fragments of the glycopeptide are loaded on major histocompatibility complex II (MHC-II) to be presented at the cell surface. A similar mechanism is applied for zwitterionic polysaccharides, however, with a different processing mechanism depending on nitric oxide (NO). Glycolipids are presented on CD1-type proteins that are similar to MHC-I after being captured by lipid transfer proteins, such as saposins. The mechanisms underlying the processing and presentation of soluble oligo- and polysaccharides are unknown but are likely to involve binding through C-type lectins expressed at the surface of antigen-presenting cells.

The pathways of antigen processing and presentation are well-established for peptide antigens. After endocytosis, peptidic antigens are broken down in phagolysosomes and fragments are presented in the groove of major histocompatibility complex (MHC)-II molecules at the cell surface (Fig. 2). MHC-II is expressed by all antigen-presenting cells, including B cells, dendritic cells, and macrophages. Intracellular antigens are processed and presented by MHC-I in a similar fashion at the surface of all cell types [69]. The production of specific antibodies results from the activation of naive B cells in association with T cells providing co-stimulatory signals. As activated B cells proliferate and enter the germinal centers of lymphoid follicles, interaction with T cells mediates class switching and somatic hypermutation and finally leads to the replacement of primary IgM antibodies with mature IgG displaying high antigen affinity. High-affinity memory B cells and long-lived plasma cells, producing large amounts of antibodies, stay in secondary lymphoid organs or migrate to the bone marrow [70, 71].

The generation of carbohydrate-specific antibodies may in part follow the classical antigen presentation pathway and T-cell-dependent activation. Glycopeptides can be presented via MHC-II like standard peptide antigens. The carbohydrate moiety can be recognized by glycan-specific B cells, while T cells specifically recognizing the same glycopeptidic antigens provide the necessary co-stimulatory activity ensuring antibody maturation [72]. Some zwitterionic polysaccharides devoid of peptidic components share the ability to be processed by MHC-II to activate T cells and B cells [73–76]. The best-studied zwitterionic carbohydrate antigens include the type 1 capsular polysaccharide from *Streptococcus pneumoniae* [74, 76, 77], the capsular polysaccharide A from *Bacteroides fragilis* [74, 77], and zwitterionic motifs in *Staphylococcus aureus* polysaccharides [78].

**Fig. 3:**
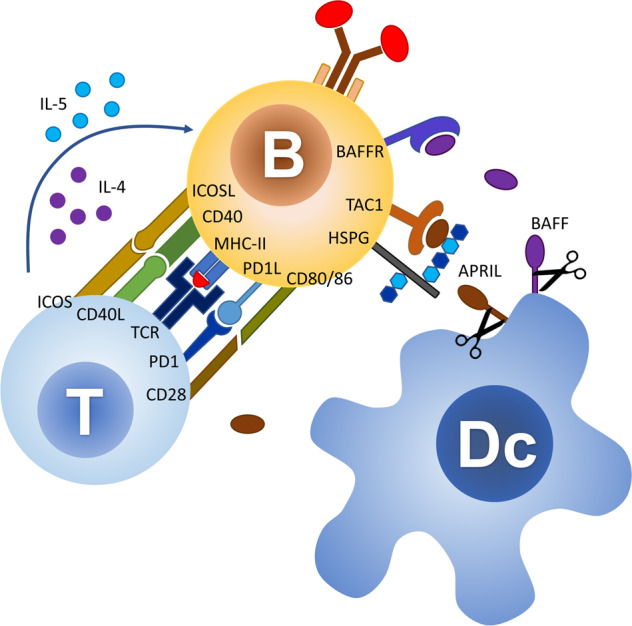
T-cell-dependent and -independent activation of B-cells. T cells activate B-cells and promote antibody class switching through multiple interactions involving antigen-bound MHC-II with the T-cell receptor (TCR), and activation though the co-receptor systems CD40-CD40L, ICOS-ICOSL, PD1/PD1L, and CD28-CD80/86. In the absence MHC-II presentation of antigens to T cells, B-cell activation and immunoglobulin class-switching can be mediated through binding to the activating proteins BAFF and APRIL secreted by myeloid cells, such as dendritic cells (Dc). APRIL binds to its receptor TAC1 on B cells after docking to heparan sulfate proteoglycans (HSPG).

Some types of antigens can activate B cells without T-cell help. T-cell-independent antigens of type 1 include heterogeneous bacterial components that function as polyclonal B-cell activators. Type 2 antigens comprise polymers with repetitive motifs, such as polysaccharides [79]. Due to their structure type 2 antigens can cross-link several B-cell receptors, thereby leading to cell activation. In many cases, T-cell-independent antigens trigger additional signals, such as binding to Toll-like receptors, to activate B cells [80]. This mechanism usually results in the generation of low-affinity antibodies of the IgM and IgG class and in the inability to stimulate germinal centers and to induce immunological memory [81]. In mucosal tissue a T-cell-independent mechanism ensures an efficient B-cell activation, featuring immunoglobulin class-switch recombination. The pathway occurs mainly in the lamina propria and isolated lymphoid follicles. B-cell activation is enhanced by the TNF superfamily proteins BAFF [82] and APRIL [83], secreted by dendritic cells, which induce the expression of activation-induced cytidine deaminase (Fig. 3). B cells activated in this way undergo class-switch recombination, but not somatic hypermutation, as they do not re-enter the germinal centers [84]. The BAFF/APRIL-mediated pathway plays a major role in the emergence of carbohydrate-specific IgG and IgA arising after the microbial colonization of the gut [85].

Glycolipid antigens can be presented to T cells by MHC-like proteins of the CD1 family that are mainly expressed on macrophages, dendritic cells, and B cells. MHC-I and CD1 are structurally related and share similar mechanisms of antigen presentation, with the exception that loading of glycolipid antigens is assisted by different lipid transfer proteins in endosomes [86]. In humans, the CD1 family consists of five isoforms, which are divided into three subgroups, CD1a–c, CD1d, and CD1e, binding different types of antigens. CD1d-presented glycolipids activate invariant natural killer T cells, which express an invariant alpha chain in their T-cell receptor, even though CD1d-reactive cells with more variable T-cell receptors have also been described [87]. CD1 isoforms are mainly associated with the presentation of glycolipids with short carbohydrate chains [88], which include microbial and self-lipids, lipopeptides, and glycolipid antigens, such as α-Gal-ceramide, a glycosphingolipid found in marine sponges [89].

Although most carbohydrate-specific antibodies are of low affinity, recent works described high-affinity antibodies recognizing *Klebsiella pneumoniae* O-antigens and related lipopolysaccharides (LPS) from other bacteria [68]. These antibodies underwent somatic mutations yielding strong antigen binding and lacked poly-reactivity. The authors suggested that the simultaneous uptake of glycan and protein antigens, both present in bacterial membranes, could explain how B cells recognizing O-antigens may indirectly receive assistance from T cells and go through affinity maturation. High levels of somatic hypermutation may result from many re-entries into germinal centers due to reactivity to different microbial species. This example underlines the importance to differentiate between unique antigen encounters, such as in nonrecurring infections, and repeated antigen exposure, as occurring in the context of commensal intestinal bacteria and environmental antigens.

## Induction of carbohydrate-specific antibodies through microbial exposure

**Fig. 4:**
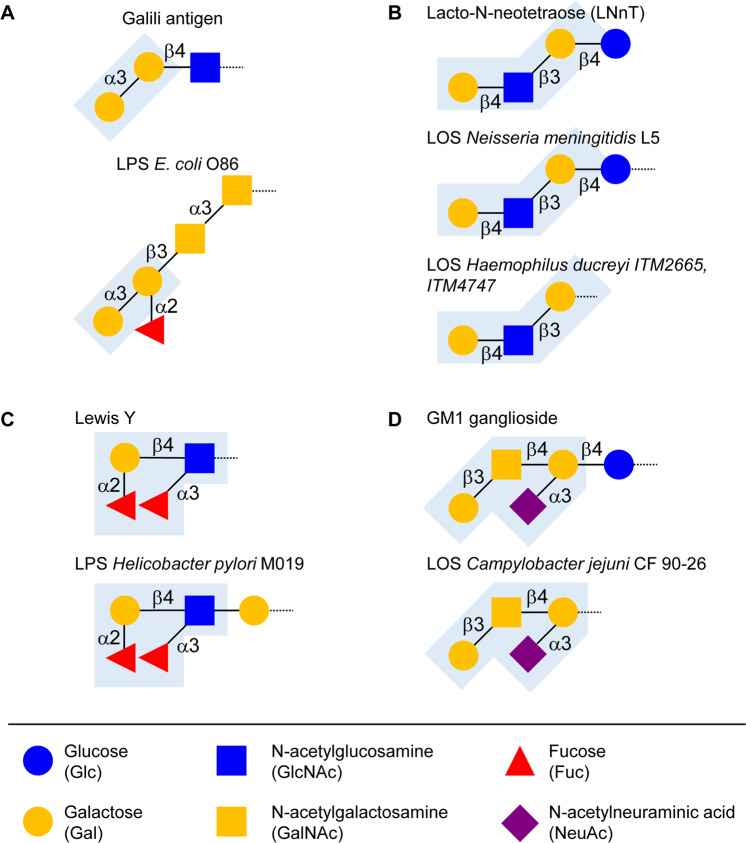
Molecular mimicry between animal glycan epitopes and bacterial glycans. **a** Schematic structure of the Galili xenoantigen and *Escherichia coli* O86 O-antigen expressed on lipopolysaccharide (LPS). The conserved Gal(α1–3)Gal motif is highlighted in blue. **b** Structures of lipooligosaccharides (LOS) of *Neisseria meningitidis* and *Haemophilus ducreyi* including the lacto-N-neotetraose (LNnT) epitope found in human milk oligosaccharides and on glycosphingolipids. **c** Similarity between the Lewis Y epitope and the LPS of *Helicobacter pylori* M019. **d** Schematic structure of the ganglioside GM1 and the LOS epitope of *Campylobacter jejuni*.

The permanent challenge of host immunity by the gut microbiota leads to high antibody titers against some carbohydrate antigens. In fact, the development of carbohydrate-specific antibodies coincides with the microbial colonization of the gut at birth. Before birth, IgG are transferred from the maternal circulation to the fetus crossing the placenta. Postnatally, additional maternal IgG and IgA are delivered through breast milk to the suckling infant [90]. Although the development and maturation of antibodies begins by the third trimester of gestation, neonatal antibodies are largely immature [91]. The development of functional IgM and IgG repertoires parallels the first contact with the extrauterine environment [92, 93], but take several years until completion of antibody maturation [94, 95]. Some carbohydrate-specific IgM have been reported in cord blood [96], although they remain marginal in the first weeks of life [97]. Within the first months of life, infants develop carbohydrate-specific antibodies such as ABO-specific IgM [98] and α-Gal antibodies [29, 99]. At the age of 8 months, infants express B1 and B2 cell-derived ABO antibodies, thus indicating the maturation of specific antibodies to these antigens beyond natural antibodies [98]. The presence of B2 cell-derived ABO antibodies underlines the antibody maturation process driven by gut bacterial stimulation [100–102]. The same principle has been suggested for the production of α-Gal-specific antibodies [25, 43]. The α-Gal epitope occurs on the surface of several *Enterobacteriaceae*, including *Klebsiella* species, *Salmonella minnesota*, and *Escherichia coli* O86:B7 [25, 103]. Colonization of mice deficient for the α1–3 Gal-transferase Ggta1 with *E. coli* O86:B7 resulted in the production of α-Gal IgM [104]. Similarly, the ingestion of *E. coli* O86:B7 in humans triggered the production of antibodies to blood group B antigen, which includes an epitope related to α-Gal [101] (Fig. 4a). A correlation between the composition of the gut microbiota and carbohydrate-specific antibodies was reported in *Ggta1*-null mice, in which changes in Clostridiales, Bacteroidales, Lactobacillales, and Deferribacterales were related to changes in the levels and repertoires of carbohydrate-specific antibodies [105].

In addition to eliciting the production of antibodies, the gut microbiota largely contributes to the development of the mucosal immune system [106–108]. Carbohydrate-specific IgA secreted into the intestinal lumen bind directly to the microbiota [109, 110]. Beyond intestinal IgA, IgG targeting gut bacterial glycans, such as LPS, are also commonly found in blood serum [111–113]. These serum IgG recognize glycan epitopes of both commensal and pathogenic bacteria [66]. The titers of serum IgG recognizing gut bacteria increase after colonization of germ-free mice [114, 115]. These systemic antibodies elicited by the gut microbiota contribute to the protection of the host against infections with *E. coli* and *Salmonella*, as shown in a mouse model [116].

Gut microbes can stimulate the production of systemic antibodies through different mechanisms. Commensal gut bacteria can reach extra-intestinal sites by direct translocation and thereby induce systemic antibody responses [117]. Diseases, such as inflammatory bowel disease and diabetes, featuring increased permeability of the intestinal epithelium show elevated levels of circulating IgG to gut bacteria [118–121]. The loss of the barrier function and a subsequent translocation of bacteria into the system generally leads to anti-commensal IgG [122]. Transient or localized changes in intestinal permeability can also be triggered by gastrointestinal tract infections, drugs, toxins, malnutrition, or even psychological stress [123, 124]. Some bacteria rely on virulent factors to penetrate the intestinal barrier and to reach the blood circulation [116]. Gut microbial antigens can also be sampled directly by dendritic cells extending their dendrites across the epithelium [125, 126]. Some microbial antigens can be captured by Peyer’s patches and processed by underlying lymphoid cells during passage through the small intestine [127].

## Roles of carbohydrate-specific antibodies

Intestinal carbohydrate-specific antibodies recognizing commensal bacteria help to control host-microbial homeostasis by mediating tolerance through the reduction of bacterial antigen expression and subsequent proinflammatory signaling [128]. Carbohydrate-specific antibodies targeting gut microbial glycans can cross-react with structurally similar antigens expressed on pathogens and thereby contribute to a broad protection against infections [44, 45]. α-Gal antibodies originally directed toward gut bacteria, for example, confer a partial protection from the malaria agent *Plasmodium falciparum*. Circulating α-Gal IgM and IgG bind to α-Gal determinants on glycosylphosphatidylinositol-anchored proteins on the sporozoite form of *P. falciparum*. This mechanism explains why people with high α-Gal IgM levels in malaria-endemic areas have a decreased risk of infection [104, 129]. In mice, α-Gal antibody levels can be increased by oral administration of *E. coli* O86:B7, which expresses the α-Gal epitope in the context of its LPS O-antigen [104] (Fig. 4a). Along this line, a gut microbiota with higher abundance of *Enterobacteriaceae*, including *E. coli* and *Shigella*, was associated with a reduced risk of *P. falciparum* infections in individuals from malaria-endemic regions [130]. The protection conferred by specific gut bacteria toward parasitic infections opens new perspectives for the incorporation of probiotics in new vaccination strategies toward malaria [131, 132].

Similar mechanisms of protection have been outlined for infections with *Trypanosoma* and *Leishmania*, as these parasites also express α-Gal on their surface glycans [133]. Accordingly, *Leishmania-* or *Trypanosoma*-infected persons produce high levels of α-Gal antibodies [131, 134, 135]. These α-Gal antibodies can lyse trypomastigotes, the extracellular form of *Trypanosoma cruzi* found in the blood of infected people [136, 137]. Recently, vaccination with α-Gal nanoparticles has been demonstrated to protect from *Leishmania* infection [138]. The protective effect of α-Gal antibodies induced after infection or immunization with glycoconjugates presenting the α-Gal epitope has been shown to be generally stronger than natural α-Gal antibodies found in healthy individuals [139]. On the other hand, in some Gram-negative bacteria, binding of LPS by α-Gal antibodies contributed to decreased complement activation through the alternative pathway, indicating that carbohydrate-specific antibodies may in some instances also contribute to the survival of pathogenic bacteria, acting so as facilitators of infectious diseases [140].

In contrast to the positive effects of α-Gal IgM and IgG in the protection from infectious diseases, α-Gal immunoglobulins of the IgE class have been associated with allergic reactions to red meat. Unclear cases of delayed anaphylactic reactions after the consumption of red meat have been associated with a history of tick bites in the affected patients [141, 142]. Further studies confirmed the induction of α-Gal IgE consecutive to tick bites [142, 143]. The α-Gal epitope was determined in the saliva of the ticks [144], but the mechanisms leading to the generation of IgE toward that carbohydrate epitope remain unclear [143, 145]. These antibodies recognize the α-Gal epitope present on glycoproteins and glycolipids of meat products from beef, pork or lamb, as demonstrated for the α-Gal epitope in beef proteins, which was recognized by IgE of meat-allergy patients [146].

Interestingly, α-Gal IgE antibodies are also responsible for triggering anaphylaxis to the anti-cancer drug Cetuximab, a monoclonal antibody containing α-Gal epitopes on its glycan chains [147]. The presence of high levels of α-Gal IgE in serum is now referred to as α-Gal syndrome and, in theory, these antibodies can provoke allergic reactions to dairy products and gelatin containing food, as well as to pharmaceuticals, antivenoms and bioprosthetic heart valves [145]. Most of the studied cases, however, are restricted to immediate allergic reactions to Cetuximab and delayed reactions to red meat or immediate reactions to innards, such as pork kidney [148]. The delayed reaction to red meat ingestion is likely explained by the slow kinetics of digestion and absorption of lipid particles carrying α-Gal-containing digestion products into the circulation [149, 150].

## Carbohydrate antigen mimicry

Despite the vast structural differences between prokaryotic and eukaryotic glycoconjugates, specific carbohydrate structures are shared between phylogenetically distant organisms. Such structural similarities can be serendipitous or purposeful. The expression of ABO blood group antigens by gut microbes likely reflects a random selection process that is unrelated to the expression of ABO antigens on human cells. Some bacteria on the other hand rely on molecular mimicry [151] in order to evade host immunity or to exploit host immunity to facilitate infection [72, 152]. Pathogenic bacteria including *Neisseria meningitidis*, *Neisseria gonorrhoeae, Haemophilus influenzae*, and *Haemophilus ducreyi* express lipooligosaccharides (LOS) carrying epitopes similar to lactoneo-series glycosphingolipids [152–157] (Fig. 4b). *N. gonorrhoeae, N. meningitidis* and *H. influenzae* also express sialylated LOS structures [158–161]. In addition to mimicking LOS, *N. meningitidis* synthesize a protective polysaccharide capsule of polysialic acid. Whereas group C meningococci produces a polymer of α2–9-linked sialic acid that is highly immunogenic, the capsule of group B meningococci is built of α2–8-linked sialic acid [72], which mimics host polysialic acid [162]. The hyaluronic acid capsule of group A streptococci is another example of antigen mimicry, which contributes to the virulence of the pathogen [163, 164] while imitating hyaluronic acid, an essential component of the human extracellular matrix [165]. Several gut bacteria express carbohydrate epitopes similar or identical to human glycans. Many strains of *Helicobacter pylori* present fucosylated O-antigens structures similar to fucosylated Lewis X or Lewis Y blood group antigens [152, 166] (Fig. 4c). Further examples of mammalian glycoprotein epitopes in bacterial O-polysaccharides are found in different *E. coli* serotypes and include T antigen, sialyl-T antigen and type 1 and 2 chains [167].

The expression of mimicking carbohydrate antigens by gut bacteria often relies on the salvage of monosaccharides derived from host glycoconjugates. *H. influenzae*, for instance, uses host-derived sialic acid to assemble its LPS [168]. *N. gonorrhoeae* also incorporates host-derived sialic acid into its LOS [160]. Fuc is another host-derived monosaccharide frequently internalized by gut bacteria. *Bacteroides thetaiotaomicron* and other members of the genus *Bacteroides* express fucosidase enzymes and Fuc transport systems enabling the internalization and utilization of the monosaccharide [169]. Besides catabolism for energy production, Fuc can be converted to the activated form GDP-Fuc by the enzyme l-fucokinase/GDP-Fuc pyrophosphorylase [170], which leads to the incorporation into bacterial capsular polysaccharides or glycoproteins [72]. While the production of mimicking carbohydrate antigens may contribute to the survival of commensal or pathogenic bacteria by enabling evasion from the host immune system, the recognition of antigens similar to endogenous glycans may lead to the generation of carbohydrate-specific antibodies triggering autoimmune reactions by cross-reacting with host glycoconjugates.

## Autoimmunity

Molecular mimicry has been shown to contribute to the emergence of autoimmune diseases, such as Guillain-Barré syndrome and multiple sclerosis. Guillain–Barré syndrome represents a typical example of autoimmune disorder that can be caused by cross-reacting antibodies emerging after a bacterial infection [171]. Guillain–Barré syndrome is a neuropathy of the peripheral nervous system, in which neurons are damaged through an immune reaction involving antibodies reacting toward surface gangliosides. The disease is a major cause of acute flaccid paralysis in humans since the elimination of poliomyelitis [172–174]. Several bacterial and viral infections have been shown to contribute to the development of Guillain–Barré syndrome. *Campylobacter jejuni*, a bacterium causing acute enteritis [175], is the pathogen most commonly associated with Guillain–Barré syndrome [176]. Antibodies directed to *C. jejuni* surface antigens cross-react with gangliosides expressed on peripheral nerves, thereby contributing to demethylation and axonal damage [177–179]. *C. jejuni* expresses sialylated LOS structures, which strongly resemble gangliosides, such as GM1 (Fig. 4d). Suspected molecular mimicry in the development of Guillain-Barré syndrome was further confirmed by studies showing anti-ganglioside antibodies in the serum of patients with Guillain–Barré syndrome [180, 181]. Evidence from animal experiments further demonstrated that rabbits immunized with ganglioside-mimicking *C. jejuni* LOS developed high titers of LOS antibodies cross-reactive with host gangliosides [182].

Multiple sclerosis is another autoimmune disease in which autoantibodies specific for carbohydrate epitopes have been identified. Autoantibodies targeting myelin proteins initiate the injury of white and gray matter of the central nervous system, thereby leading to progressive muscle weakness, paresthesia, vision changes and cognitive decline [183, 184]. Increased levels of antibodies targeting the gangliosides GM1, GM2, and G7 have been reported in multiple sclerosis patients [185, 186]. Additional autoreactive carbohydrate-specific antibodies have been identified over the past years, such as antibodies reacting with Glc(α1–4)Glc [10, 187], galactocerebroside [188], and sulfated carbohydrates [189]. The involvement of possible infectious agents in the development of these autoreactive antibodies remains unclear.

Increased titers of carbohydrate-specific antibodies also occur in inflammatory bowel disease, which comprises Crohn’s disease and ulcerative colitis. These antibodies recognize oligosaccharides that are frequently found on the surface of gut microbes, such as laminaribioside, laminarin, mannobioside, chitobioside, and chitin [190–193]. In our own studies, we observed an increased antibody response to carbohydrates in blood serum of patients with Crohn’s disease compared with healthy controls, which was mainly reflected by a higher antibody reactivity to fucosylated oligosaccharides and could be linked to increased antibody recognition of intestinal *Bacteroides* species [194]. Given the association between intestinal dysbiosis and altered anti-bacterial antibodies, the question arises whether these antibodies targeting bacterial antigens contribute to the etiology of inflammatory bowel disease. Despite the large number of studies outlining alterations of the intestinal microbiota and increase of carbohydrate-specific antibodies in inflammatory bowel disease, a direct connection between these antibodies and an exacerbation of the inflammatory response observed in inflammatory bowel disease is still to establish. Beyond the examples addressed here above, increased levels of carbohydrate-specific antibodies have been also reported in other autoimmune diseases, including systemic lupus erythematosus, diabetes type 1 and post-streptococcal heart disease [186, 195–198].

## Analytical tools

The investigation of carbohydrate–protein interactions significantly lags behind similar studies on other macromolecule interactions because of the challenges associated with the purification and synthesis of glycans. Considering the broad structural diversity of glycoconjugates, techniques enabling the parallel and quantitative analysis of a wide range of carbohydrate structures are likely to yield the most reliable information about the specificity and affinity of antibodies recognizing glycan antigens. Accordingly, large-scale glycan arrays have turned out as being valuable resources for the quantitative analysis of carbohydrate–protein interactions [199].

While arrays displaying nucleic acids [200] have been applied widely for several decades, the first glycan arrays [201–203] emerged in the early 2000s. Glycan arrays mainly consist of purified or chemically synthesized glycans, which are immobilized on glass slides by either direct adsorption of glycans to nitrocellulose surfaces or different methods of chemical linkage. Using glycans with attached chemical linkers and slides covered with matching functional groups, covalent immobilization chemistry overcomes the limitations of adsorption, which include varying immobilization efficiencies and unspecific binding. Chemical immobilization is mostly performed by reaction of amine-functionalized glycans with N-hydroxysuccinimide esters, thio-functionalized glycans with maleimide groups and amine- or thio-functionalized glycans with epoxy groups. In addition, glycans without a linker can be coupled covalently via the free reducing end to surfaces with hydrazides or oxyamines [204, 205]. One type of arrays displays known structures, whereas shotgun arrays are made up of glycans, isolated from natural sources with yet undefined structures. Besides the structural analysis of single unknown glycans by mass spectrometry and NMR spectroscopy, glycans on shotgun arrays can be analyzed by glycan-binding proteins directly on the array [206]. Glycan arrays with defined glycan structures have been applied to characterize the binding specificity of glycan-binding proteins, where previously reported specificities of several plant lectins, human, bacterial, and viral glycan-binding proteins could be confirmed [207]. The same study detected a variety of carbohydrate-specific antibodies in human serum and suggested the use of glycan arrays in diagnosis [207]. Indeed, glycan arrays are also used as diagnostic tools to detect carbohydrate-specific antibodies targeting glycan structures specific for bacterial or viral antigens or to glycan cancer markers. An advantage of the detection of immobilized well-characterized polysaccharides by human serum antibodies over the detection with crude bacterial lysates is the avoidance of false-positive diagnoses [208]. The specific detection of antibodies to capsular polysaccharides of *Burkholderia mallei* in the serum of a human glanders patient compared with the absence of these antibodies in the pre-infection serum of the same person was demonstrated [208]. Further, microarray analysis allowed for the differentiation of different types of *Salmonella* infections. Testing sera from patients with verified types of salmonellosis on a microarray with O-antigenic oligosaccharides specific for *Salmonella enterica* serovar Paratyphi, Typhimurium, or Enteriditis, antibodies were correctly detected [209].

The application of glycan arrays can provide valuable information complementary to established genetic markers, for example when establishing new biomarkers in different types of cancer. Based on glycan arrays, 24 glycans could be identified that significantly discriminated between malignant tumors and healthy controls in the context of ovarian cancer [210]. Another study showed the association between serum antibody levels to specific glycans in Hodgkin’s lymphoma [211]. Glycan arrays are also used in the development of glycoconjugate vaccines, by enabling the quick screening of a broad panel of potential glycan structures as targets for serum antibodies from infected people [212]. For example, the analysis of stool and serum samples from *Clostridium difficile* patients on microarrays containing oligosaccharide epitopes of the *C. difficile* cell wall polysaccharides PS-I and PS-II confirmed novel vaccine candidates [213]. Glycan arrays are powerful resources when assessing bacteria or viruses binding to host carbohydrate structures, such as interactions of the influenza virus to host receptors [214–217]. Further, arrays consisting of immobilized antibodies or lectins have been developed to analyze carbohydrate-binding properties [218]. Additional tools to analyze carbohydrate-specific antibodies in a similar fashion include classical methods, such as ELISA [219, 220], but also innovative approaches, including multiplex suspension arrays [221] or cell-based glycan arrays [222].

Another method to study unknown glycans structures is the analysis of enzymes required for glycan synthesis, such as glycosyltransferases or transport proteins for glycan precursors. Given that glycans, in contrast to proteins, are not encoded by a genomic template, they cannot be directly identified at the genomic level. The identification of genes encoding glycosyltransferases or transport proteins for glycan precursors yields information to predict glycan assembly. Recently, gene analysis identified thousands of glycan enzymes in *Bacteroides* species [223]. In bacterial genomes, glycosylation genes are often clustered in glycosylation loci, which can be identified by searching for conserved genes. Identified genes can be expressed recombinantly to verify their function and to analyze resulting glycan structures. Additional genetical engineering of commensal bacteria using CRISPR interference to manipulate gene expression was introduced recently [224]. Approaches with CHO cells or engineered phage display for glycan arrays combine the genomic possibilities with classical glycan array analysis to allow fast and cost-effective high-throughput testing [222, 225]. Cell-based glycan arrays enable testing of glycan binding directly on the surface of CHO cells by flow cytometry. Using recombinant glycosyltransferases, sialic acid, and Fuc, the small number of different glycan structures naturally occurring on CHO cells can be transformed into a diverse glycan repertoire with distinct carbohydrate epitopes. The utility of cell-based glycan arrays was demonstrated by the identification of high-affinity ligands linked to osteoclast differentiation when testing osteoprogenitor cells with cells expressing Siglec-15 ligands [222]. To overcome the challenges of chemical synthesis or time-consuming isolation of glycans, glycoarrays with engineered *E. coli*-derived phages displaying diverse surface glycan epitopes have been developed. In contrast to cell-based glycan arrays, where cells are detected by flow cytometry, glycophages can be immobilized on glass slides, enabling high-throughput detection of glycan interactions in a classical array format [225].

## Concluding remarks

Carbohydrate-specific antibodies are often referred to as natural antibodies without underlying specific maturation process. This simplistic view does not account for the large number of carbohydrate-specific antibodies emerging in response to the exposure to the gut microbiota starting at birth, as demonstrated for the high titers of ABO- and α-Gal-specific antibodies. The presence of systemic bacterially induced carbohydrate-specific antibodies contributes to the protection from pathogens but can in cases of antigenic mimicry also account for the development of autoimmune diseases. The functions of carbohydrate-specific antibodies largely underline their significance in human health and disease.
